# Efficacy of Extended Valganciclovir Prophylaxis in Preventing Cytomegalovirus Infection in Pediatric Kidney Transplantation

**DOI:** 10.2174/1874303X01407010152

**Published:** 2014-12-31

**Authors:** Faris Hashim, Jon A. Gregg, Vikas R. Dharnidharka

**Affiliations:** 1 Divisions of Transplant Nephrology, University of Florida College of Medicine, Gainesville, FL USA; 2 Pediatric Nephrology and Transplant Nephrology, University of Florida College of Medicine, Gainesville, FL USA

**Keywords:** Late onset CMV infection, renal transplant, short term and long term prophylaxis, valgancyclovir.

## Abstract

Cytomegalovirus (CMV) is one of the most frequent opportunistic infection in renal transplant (RTx) recipients. Valganciclovir (VGC) has been showed to be safe and highly effective in prophylaxis of CMV infection in RTx recipients. Recently, an increase in delayed onset CMV disease has been noted with some arguing that longer prophylaxis may decrease the late-onset disease.

We retrospectively tested the hypothesis that extended term prophylaxis (ETP) of VGC for 12 months is more effective than short term prophylaxis (STP) of 6 months in preventing CMV infection and disease in pediatric RTx performed at the University of Florida from July 2003 to December 2010. In this period, all recipients underwent prospective CMV PCR (Polymerase Chain Reaction) monitoring and were maintained on similar immunosuppression.

Eighty six patients received RTx during that period. All eligible subjects had to have at least 12 months of graft survival and 18 months of follow up, leaving 73 eligible subjects in final study group. CMV infection or disease occurred in 6/29 (20%) in the STP group vs 6/44 (14%) in the ETP group with no statistical significant difference (P= 0.42). Donor positive/recipients negative CMV serology status (D+/R-) were associated with a higher risk of CMV infection in both univariate and multivariate analysis (P=0.01). Anemia and Leucopenia directly associated with VGC were similar in both groups (P=0.58 and P=0.2 respectively). Biopsy-proven acute rejection was also non-significant in both groups (P=0.39).

Although ETP for CMV from 6 months to 12 months is safe and has minimal adverse effect, it did not reduce CMV infection or disease. Further controlled studies in pediatrics age group are considered to compare longer versus shorter periods of prophylaxis and their impact on prevention of CMV infection, resistance, cost, and toxicity.

## INTRODUCTION

The more potent immunosuppressive therapy that has successfully reduced the incidence of acute rejection, has also resulted in a higher incidence of viral infection [1]. In the pediatric RTx population, infections have replaced rejection as the leading cause of hospitalization [2]. CMV is one of the most important infections in RTx recipients, exposure to the virus increases with age in the general population and is present in more than two-thirds of donors and recipients prior to transplantation [3]. CMV can cause either CMV disease (fever, malaise and cytopenia) or subclinical viral infection usually in the first year post solid organ transplant [4]. CMV may also increase the risk for other opportunistic infections, rejection, and long-term allograft dysfunction [1, 5].

Prevention and treatment of CMV primary infection or reactivation has become increasingly important in the management of this patient population. More recently, VGC has become the standard drug used for prophylaxis against CMV infection [6]. Currently, two different strategies are commonly used to prevent this disease in adult patients: prophylaxis; which is administered during the first 3-6 months following transplant for CMV D+/R- and CMV R+ with no prophylaxis is recommended for CMV D-/R-, or preemptive therapy, which is commenced as soon as viremia is detected through periodical laboratory tests. Since the introduction of these two strategies, an increase in the incidence of late-onset CMV disease has been noted [7, 8].

The IMPACT study is the largest study to demonstrate the effectiveness of prolonging prophylaxis from 3 to 6 months in adult RTx recipients in improving CMV disease up to 12 months post-transplant. Other recent supportive studies performed in adult lung transplant recipients, demonstrated that 12 months of CMV prophylaxis is beneficial compared to 3 months [9-11].

Although adult and pediatric patients share similar risk factors for developing post RTx CMV disease, the pediatric population has a higher frequency of high-risk patients given the greater proportion of recipients with negative CMV serology to seropositive donors. Despite this situation, very few studies have been carried out in children, and management strategies are for the most part based on results from the adult population [12, 13]. Only one pediatric study so far showed that 24 weeks of prophylaxis was associated with a lower rate of late onset disease than previous reports with 12 weeks regimens, the prolong treatment was also safe and without a significant increase in toxicity and resistance [14].

## METHODS

After approval by the University of Florida Institutional Review Board, we retrospectively evaluated the electronic records for Pediatric RTx recipients between the ages of one to 18 years who underwent RTx at Shands/UF between July 1, 2003 and December 31, 2010. All eligible subjects had to have at least 6 months of graft survival and 18 months of follow up, otherwise those cases were censored out. Data collected from both groups include demographics, immunosuppressive, VGC duration, CMV disease, acute rejection, and hematologic adverse events.

At our center most recipients received induction therapy with rabbit anti-thymocyte globulin (Thymoglobulin, Genzyme, Cambridge, Massachusetts, USA; daily doses of 1.5 mg/kg/day for 3 days with total dose of 7.5 mg/kg). Maintenance medication included calcineurin inhibitor (tacrolimus or cyclosporine) with the dosing adjusted according to the whole blood trough level, mycophenolate mofetil (MMF 600 mg/m2 twice daily standard pediatric dose and 750 mg/ m2 twice daily for AA children). In case we used mycophenolate sodium 400mg/m2 were used. Delayed graft function was defined as hemodialysis within one week post kidney transplant. Prophylactic valganciclovir hydrochloride (Valcyte; Hoffmann-La Roche, New Jersey, USA) was given at a dose of 10-12 mg/kg/day max 900 mg. The dose was adjusted according to the calculated glomerular filtration rate (GFR) by Schwartz equation. All recipients were given VGC for either 6 months STP or 12 months ETP after RTx. If a patient required thymoglobulin for treatment of rejection after the time of prophylaxis, an additional 3 months of prophylactic VGC administration post thymoglobulin was given.

Surveillance for CMV infection was done with PCR once monthly for the first 12 months post RTx. All PCR assays were performed at Shands hospital clinical laboratories. Any level of detection was considered positive. Confirmation of CMV infection was defined by detection of CMV PCR in plasma on 2 consecutive positive plasma samples within 2 weeks and we considered disease to be present when the virus detection was supplemented by viral syndrome including fever >38cº, myalgias, and arthalgias with or without accompanying symptoms of specific organ involve-ment (hepatitis, pneumonitis, colitis or meningoencephalitis). Treatment of symptomatic infection was done with intravenous (IV) ganciclovir 5-6 mg/kg given every 12 to 24 hours for 2 weeks and for asymptomatic infection is by using a treatment dose of VGC 15 to 18 mg/kg given orally twice daily for 2 weeks and then keep the patient on prophylactic dose for an extra coarse of 3 months. Anemia was defined as hemoglobin <10 g/dl and leukopenia as white blood cells count <3000 cell/mcL. Acute rejection was defined as biopsy-proven rejection, acute cellular rejection treated with IV methylprednisolone or thymoglobulin depending on the severity.

## Statistical Analysis

Recipients’ demographics and clinical events were identified by reviewing medical records, clinical transplant databases, clinical laboratory data, and pathology reports. Data were entered to SAS Statistic software, version 9.3 (Cary, NC, USA). Results are expressed as proportion (%) or mean (standard deviation; SD). Analyses were performed with Chi-Square testing for categorical variables (Fischer’s exact tests as appropriate) and student's t-test for continuous variables (Mann-Whitney test was used for non-normally distributed variables), respectively.

Time to CMV viremia was calculated as the time between transplant and laboratory confirmation of CMV Viremia. Statistical significance for univariate comparisons was defined as p<0.05. Multivariate stepwise logistic regression models were fitted to study the adjusted relationship between the CMV infection and several covariates including risk group, gender, RTx type, induction medication and graft function.

## RESULTS

One thousand and thirty-two blood samples were drawn from 86 patients who received RTx from July 2003 to December 2010. All eligible patients had to have at least 12 months of graft survival and 18 months of follow up, leaving 876 samples from 73 eligible patients in the final study group. Median age at Rtx was 12 years (range 1-17 yrs) in the STP group and 11 years (range 2-17 yrs) in ETP group. Median duration of VGC use in the STP group was 6 months (range 6-7) and 12 months (range 11-13) in the ETP group. As expected, 41/68 (60%) of donors (5 missing cases) and 30/73 (41%) of recipients were seropositive pre transplant, a total of 30 patients (41%) were considered at high risk (D+/R-) for CMV infection (30% from ST *vs* 51% ET group).

Notable features of the differences in the demographic feature in this population include longer duration of VGC prophylaxis in those with delayed graft function and positive CMV serostatus. Detail of patient demographics in both groups and donor (D)/recipient (R) CMV serostatus were summarized in Table **1**.

During the study period, CMV viremia or disease occurred in 6/29 (21%) in the ST group *vs* 6/44 (14%) in the in the ET group (P=0.42), Table **2**. One recipient from each group developed systemic CMV while they were on prophylaxis and responded well to the IV ganciclovir treatment for 2 weeks with improving of symptoms and gradual disappearance of viremia. No grafts were lost to systemic CMV disease. All asymptomatic infection also responded well to 2 weeks of treatment dose of VGC and no resistance was reported to therapeutic oral VGC despite previous prophylaxis.

Being in the high risk CMV serology group (D+R-) was associated with a significantly higher risk for CMV infection or disease in both groups (Table **3**). This is also confirmed in a multivariate logistic regression model (Table **4**). Anemia, leucopenia and biopsy proven acute rejections were not significant in both groups (Table **5**).

## DISCUSSION

CMV continues to be an important cause of morbidity and mortality in children who have received RTx. Children have an increased likelihood of acquiring primary CMV infection because they are more often CMV negative at the time of surgery. However, the duration of prophylaxis is still an area of debate. Consensus recommendations guide the duration of therapy and have recommended 3-6 months of prophylaxis based on the serostatus of the donor and recipient [15, 16].

Despite many studies focusing on the pediatric organ transplant population, standard protocols for CMV monitoring and administering antiviral therapies are not established in this population [17-20]. In 2011, Camacho-Gonzalez, *et al.* showed that prolonging VGCV prophylaxis to 6 months is safe and decreases the incidence of late onset CMV disease without a significant increase in toxicity and resistance [14]. However, the study was limited because of the fact that they did not have a control group. Our study is the first study to compare the efficacy of prolonged (12 moths) VGC prophylaxis in preventing CMV infection and disease in the pediatric RTx population.

Strict cut-off values of CMV PCR indicating need of therapeutic intervention are not available. Values around 1000-2000 IU/mL have been suggested [21, 22]. Our hospital policy is to consider any level of detection as positive which will increase the sensitivity of the test. Despite that, our data revealed an overall rate of CMV infection of 16% (21% from ST group versus 14% from ET group). These results are lower than what has been reported in other previous pediatric studies with less duration of prophylaxis [8, 14, 18, 23]. Humar *et al.* in their prospective randomized controlled trial showed a cumulative incidence of 21% in adult RTx recipient who received 200 days of VGC prophylaxis [9]. However, no real comparisons can be made between our study and this study as they are completely different in the design and prophylactic approach.

Our results did not show any significant difference between the ETP and STP in reducing the CMV infection and disease (P=0.42). Nevertheless, the multivariate logistic regression model has been showed (in accord with prior studies) that CMV serology group (D+/R-) was associated with significantly higher risk for developing CMV infection or disease in both group (P = 0.01)[9, 14, 23, 24].

None of our patients who developed CMV infection during prophylaxis were unresponsive to therapy with oral VGC or IV ganciclovir despite previous prophylaxis which indicate less risk of developing drug resistance despite the prophylaxis therapy. Lastly, we did not observe significant differences in presumed treatment related adverse events between the two treatment groups. This signifies the safety of VGC as a prophylaxis treatment in renal Tx pediatric population, similar to results from several previous studies [14, 25].

To summarize, our results demonstrate that although a 12-month regimen of oral VGC was a safe approach for long-term prevention of CMV infection in RTx pediatric recipients, it did not show a significant difference compared with 6 months of therapy.

## Figures and Tables

**Table 1.** Demographic and clinical characteristic, by treatment group.

Demographic Variable Total No.73	STP (6 Months) N= 29 (40%)	ETP (12 Months) N= 44 (60%)	P Value
Average age group, n (%) 0 - <6 yr 6 - <12 yr 12 - <18 yr	4 (14%) 7 (24%) 18 (62%)	6 (14%) 17 (39%) 21 (48%)	0.40
Gender, n (%) Male Female	20 (69%) 9 (31%)	28 (64%) 16 (36%)	0.60
Race, n (%) White African-American Others	13 (45%) 13 (45%) 3 (10%)	31 (70%) 12 (27%) 1 (3%)	0.24
Induction, n (%) Thymoglobulin Simulect Others	21 (72%) 1 (3%) 7 (24%)	33 (75%) 3 (7%) 8 (20%)	0.49
Recipient CMV serology, n (%) Positive Negative Donor CMV serology, n (%) Positive Negative Missing	12 (41%) 17 (59%) 13 (45%) 15 (52%) 1 (3%)	18 (40%) 26 (59%) 28 (64%) 12 (27%) 4 (9%)	0.57 0.05
Graft function post RTx, n (%) Immediate GF Delayed GF	27(93%) 2 (7%)	32 (73%) 12 (27%)	0.03
Transplant type, n (%) Living donor Deceased	11 (38%) 18 (62%)	9 (20%) 35 (80%)	0.06
Rejection, n (%) Yes No	2 (7%) 27 (93%)	8 (18%) 36 (82%)	0.17

**Table 2.** Details of CMV infection by treatment groups.

Demographic Variable Total No.73	STP (6 Months) N= 29 (40%)	ETP (12 Months) N= 44 (60%)	P Value
CMV infection, n (%) Yes No	6 (21%) 23(79%)	6 (14%) 38 (86%)	0.42

**Table 3.** CMV serology and CMV infection by treatment group infection.

Positive CMV Serology	STP (6 Months)	ETP (12 Months)	Fisher’s Exact Test
High Risk Group (D+/R-) Intermediate and low Risk (D+/R+ & D-/R+)	3/6 (50%) 3/6 (50%)	6/6 (100%) 0/6 (0%)	0.01

**Table 4.** Logistic model for the CMV infection.

Predictor Variable	P Value
Prophylactic VGC	0.42
High risk group(D+R-)	0.01
Gender	0.56
Tx type	0.80
Induction medication	0.43
Graft function	0.41

**Table 5.** VGC side effects and acute rejection by treatment group.

Total No. 73	STP Group (6 Months) 29 (40%)	ETP Group (12 Months) 44 (60%)	P Value
Anemia Yes No	6 (21%) 23 (79%)	8 (18%) 36 (82%)	0.58
Leukopenia Yes No	2 (7%) 27(93%)	7 (16%) 37 (84%)	0.20
Rejection post RTx Yes No	5 (17%) 24 (83%)	8 (18%) 36 (82%)	0.39

## LIMITATION

Although our study was limited because of its retrospective nature and the fact of the small sample size comparing to the adult studies, it’s the only study that evaluated the efficacy and safety of 12 months VGC prophylaxis comparing to the other strategy in RTx pediatric patients. In addition, the study was a single center study which might limit the generalizability of the study result. Multicenter, randomized, clinical studies involving several pediatric transplant centers are needed to help better define optimal duration of CMV prophylaxis in the pediatric age group.

## ABBREVIATIONS

CMV =: Cytomegalovirus
RTx =: Renal Transplant
STP =: Short term prophylaxis
ETP =: Extended term prophylaxis
VGC =: Valganciclovir
PCR =: Polymerase chain reaction
